# Vertigo and Ischemic Stroke after Hyperextension (Beauty Parlour Stroke syndrome)

**DOI:** 10.15388/Amed.2022.29.2.2

**Published:** 2022-06-29

**Authors:** Bünyamin Tosunoğlu, Sultan Merve Ünal, Seyfi Emre Aksoy, Tahir Kurtuluş Yoldaş

**Affiliations:** Ankara Education and Research Hospital, Department of Neurology, Ankara, Turkey; Ankara Education and Research Hospital, Department of Neurology, Ankara, Turkey; Ankara Education and Research Hospital, Department of Neurology, Ankara, Turkey; Ankara Education and Research Hospital, Department of Neurology, Ankara, Turkey

**Keywords:** Stroke, beauty parlour, trauma

## Abstract

Beauty parlour stroke syndrome is a stroke syndrome that occurs secondary to hyperextension of the neck due to compression of the vertebral artery in the atlanto-occipital region. It was first defined as “pearl beauty stroke syndrome” in 1992 by Weintraub et al. Vertigo syndrome caused by cervical region pathologies, bad posture of the neck and/or trauma [1]. We present a young, 23-year-old patient who has no disease, no trauma history nor substance-drug use. He was diagnosed with vertigo that started after going to the barber’s and then had an infarction in the cerebellum.

## Santrauka.

Grožio salono insulto sindromas yra insulto sindromas, kuris atsiranda po kaklo hiperekstenzijos dėl stuburo arterijos suspaudimo atlantinėje pakaušio srityje. 1992 m. pirmą kartą jį kaip grožio salono insulto sindromą apibrėžė Weintraub ir kt. Vertigo sindromą sukelia gimdos kaklelio srities patologija, bloga kaklo laikysena ir (arba) traumos [1]. Mūsų 23 metų jaunam pacientui, neturinčiam jokių rimtų ligų, patirtų traumų ir nevartojančiam narkotikų, buvo diagnozuotas galvos svaigimas, prasidėjęs po lankymosi kirpykloje, jį ištiko smegenėlių infarktas.

## Introduction

Vertigo refers to a feeling of dizziness or spinning. Vertigo can be a result of a particular neck posture or movement. Poor neck posture, neck disorder or cervical spine trauma causes this condition. Vertigo is usually caused by head trauma that disrupts the head and neck alignment [2,5,6]. This dizziness often occurs after moving the neck and can also affect the sense of balance and concentration.

There are a number of potential causes for vertigo, but this condition is still under investigation. Some of the causes are occlusion of the arteries in the neck due to hardening (atherosclerosis) or rupture of these arteries (dissection). In these cases, the dizziness is caused by the interruption of blood flow to the inner ear or lower brain region called the brain stem. Arthritis, surgery or trauma to the cervical region can also block the blood flow to these important areas what may result in a type of vertigo, too [2,6,7], It has also been reported that vertebrobasilar ischemia may develop during the rotation and extension movements of the cervical spine. The vertebral artery may be compressed with the rotation of the head due to its proximity to the bone, muscle and ligament in the cervical region.

In this report, we present a young patient who experienced vertigo symptoms due to neck hyperextension and then had an ischemic stroke.

## Case report

A 23-year-old male patient who had no previous dizziness and/or vertigo symptoms was admitted to the emergency department with complaints of severe dizziness, nausea and vomiting.

He had no known disease and used neither medication nor substance. Also no trauma history was reported. He said that he went to the barber shop three-four days earlier and felt a pain in his nape of the neck after putting his neck in the hyperextension position while the barber was shaving.

In his physical examination, fever was 36.6 °C, blood pressure was 120/80 mmHg, heart rate was 80/minute, respiration was 19/minute, and oxygen saturation was 98. In his neurological examination, he was conscious, oriented and cooperative. His pupils were isochoric, light reflex was observed in both eyes, and horizontal nystagmus was present in his right eye. His speech was normal and there was no facial asymmetry. On motor examination, there was no loss of strength in the upper and lower extremities. Sensory examination was normal, deep tendon reflexes were normoactive, and plantar response was normoactive. There was no sign of meningeal irritation. In the cerebellar examination, the finger–nose test was clumsy on the right. He had right ataxia in his gait.

The patient was admitted to our neurology service for further examination and treatment.

**Figure 1.**
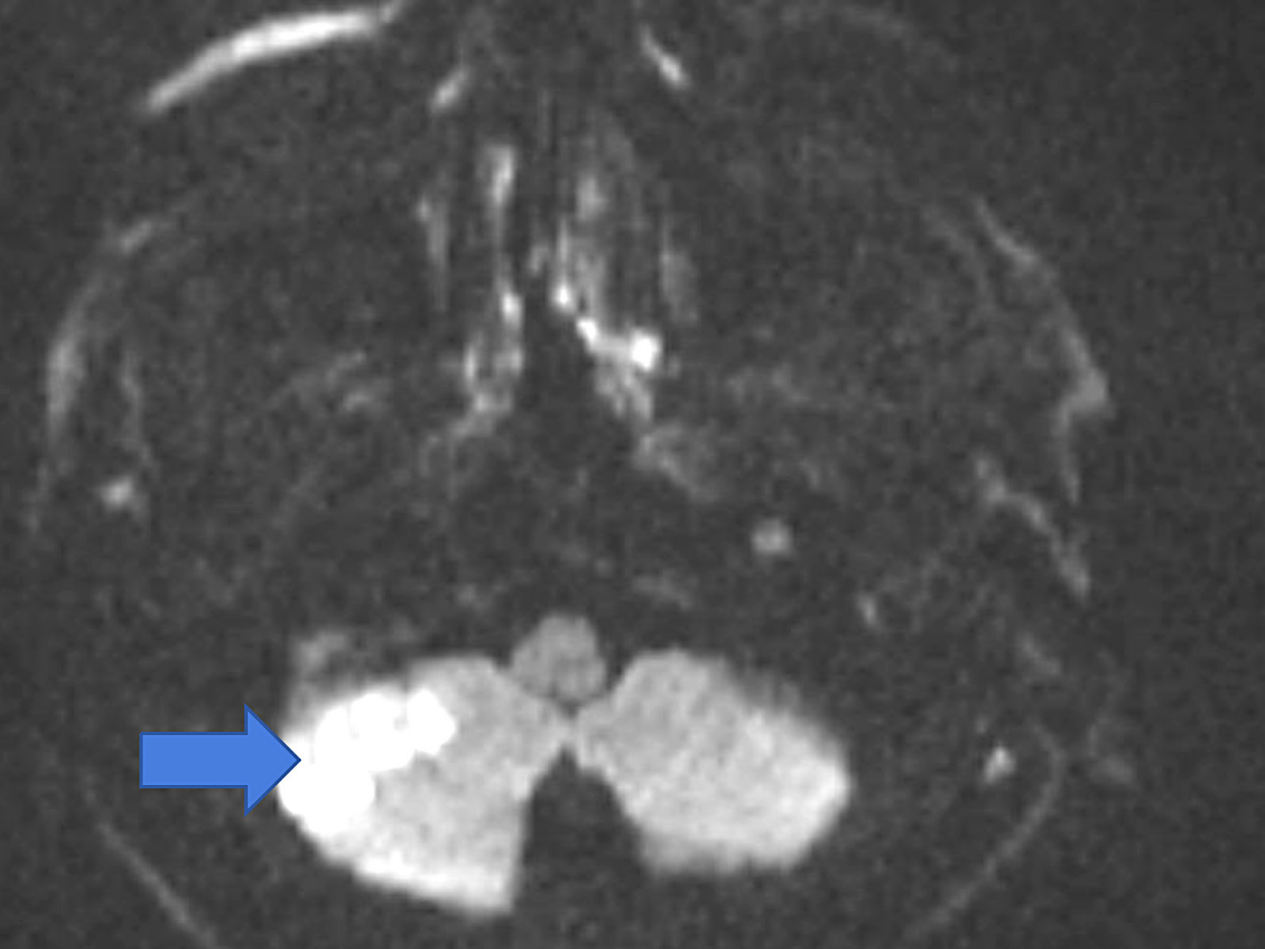
Diffusion MRI – right cerebellar infarct

The results of routine blood, biochemistry, whole blood and vitamin B12 tests were normal. Elisa tests were negative. Complete urinalysis was within normal limits. Results of HLAb5 tests for vasculitis, thrombophilia, Fabry and Behçet’s were normal. No acute pathology was detected in the brain computed tomography (CT). Magnetic resonance imaging (MRI) revealed diffusion restriction in the right cerebellum (Figure 1).

Magnetic resonance angiography revealed the blood flow disorder at the vertebral artery level at the right atlanto-occipital junction. The patient was diagnosed with the cerebellar infarction secondary to neck hyperextension and was treated conservatively with acetylsalicylic acid 300 mg 1×1 and omeprazole 1×1. Within nine days his gait and cerebellar signs improved. He was recommended neurology follow-up and discharged at the end of the tenth day.

## Discussion

In this case, cerebellar infarction occurring after cervical vertigo secondary to hyperextension is presented.

Vertigo can be a syndrome that occurs secondary to neck posture and pathologies and/or trauma [3]. While the vertigo is diagnosed, there should be no hearing symptoms (out of tinnitus) or hearing loss, but may be an ear pain (otalgia). Beauty salon stroke syndrome was first described in 1992 by Weintraub et al. When the literature is reviewed, it has been revealed that a complete anamnesis is necessary so that this diagnosis is not overlooked [1,4]. The symptoms at the time of our case can occur in many diseases. Routine radiography, computed tomography (CT), and magnetic resonance imaging (MRI) studies are often not helpful in identifying lesions in this syndrome [2,4]. Magnetic resonance angiography findings are confirmatory. The most probable pathophysiological mechanism in beauty salon stroke syndrome is vertebral stenosis due to compression of the vertebral artery at the atlanto-occipital junction [3,5,6]. However, Thiel et al. found no occlusion in the vertebral artery in their study and attributed this to decreased blood flow secondary to hyperextension of the vertebral artery. As a result, a good history should be taken in patients presenting with similar symptoms, and the employees of beauty salons, barbers and hairdressers should be informed about the related syndrome while receiving vocational training.
